# The Markers of Glutamate Metabolism in Peripheral Blood Mononuclear Cells and Neurological Complications in Lung Cancer Patients

**DOI:** 10.1155/2016/2895972

**Published:** 2016-12-04

**Authors:** Slawomir Michalak, Joanna Rybacka-Mossakowska, Wojciech Ambrosius, Joanna Gazdulska, Iwona Gołda-Gocka, Wojciech Kozubski, Rodryg Ramlau

**Affiliations:** ^1^Department of Neurochemistry and Neuropathology, Poznan University of Medical Sciences, Poznan, Przybyszewskiego str. 49, 60-355 Poznan, Poland; ^2^Department of Neurology, Poznan University of Medical Sciences, Przybyszewskiego Str. 49, 60-355 Poznan, Poland; ^3^Department of Chemotherapy, Wielkopolska Cancer Center, Garbary Str. 15, 61-866 Poznan, Poland; ^4^Sub-Department of Diurnal Chemotherapy Wielkopolska Center of Pulmonology and Thoracosurgery of Eugenia and Janusz Zeyland, Szamarzewskiego 62, 60-569 Poznan, Poland; ^5^Department of Oncology, Poznan University of Medical Sciences, Poznan, Szamarzewskiego Str. 82/84, 60-569 Poznan, Poland

## Abstract

*Objective*. To evaluate the involvement of glutamate metabolism in peripheral blood mononuclear cells (PBMC) in the development of neurological complications in lung cancer and during chemotherapy.* Methods*. The prospective study included 221 lung cancer patients treated with chemotherapeutics. Neurological status and cognitive functions were evaluated at baseline and after 6-month follow-up. Glutamate level, the activities of glutaminase- (GLS-) glutamate synthetizing enzyme, glutamate dehydrogenase (GDH), and glutamate decarboxylase catalyzing glutamate degradation were analyzed in PBMC and in sera of lung cancer patients by means of spectrophotometric and colorimetric methods.* Results*. Chemotherapy of lung neoplasms induced increase of glutamate content in PBMC and its concentration in serum increased the activity of GDH in PBMC and decreased activity of glutaminase in PBMC. The changes in glutamate metabolism markers were associated with initial manifestation of neurological deficit in lung cancer patients and with new symptoms, which appear as a complication of chemotherapy. Moreover, the analyzed parameters of glutamate control correlated with a spectrum of cognitive functions measures in lung cancer patients.* Conclusion*. We have demonstrated dysregulation in glutamate and glutamate metabolism controlling enzymes as promising indicators of risk for chemotherapy-induced neurological complications in lung cancer patients with particular emphasis on cognitive impairment.

## 1. Introduction

Glutamate is a dicarboxylic amino-acid, which plays pleiotropic role as a metabolic molecule, neurotransmitter in the nervous system, signaling agent in nonneural tissues, and pain signal transducer [1]. In the central nervous system (CNS) glutamate is an abundant excitatory amino-acid present in all neurons [2]. In pathological conditions elevated glutamate level leads to excitotoxicity [3], which is the injury of neurons caused by excessive activation of glutamate receptors. Glutamate excitotoxicity is observed in a spectrum of acute CNS pathologies including ischemia [4], traumatic brain injury [5], and status epilepticus [6]. It is hypothesized that glutamate excitotoxicity can play a role in amyotrophic lateral sclerosis [7], Huntington [8], and Alzheimer's disease [9]. During the course of neurodegenerative disorders impairment of cognitive functions belongs to the spectrum of clinical symptoms and glutamate related disturbances play an important role [10].

There are a considerable number of observations of cognitive dysfunction in lung cancer patients. Deficits in at least one of neurocognitive tests appear in almost 50% of lung cancer patients, near 40% show impairment in executive functions, learning, and memory, and 30% have slowed processing speed or present motor coordination disturbances [11]. Cognitive impairment is considered in lung cancer patients as a predictor of unfavourable outcome, for example, after radiation therapy of lung cancer patients [12]. Chemotherapy complications in CNS known as “chemobrain” or “chemo fog” manifest as cognitive impairment [13] and significantly affect activity of daily living in oncological patients. In our previous study [14] we have found that upregulation of mitochondrial activity is associated with cognitive deterioration in lung cancer patients.

The role of glutamate dysregulation in the development of cognitive disturbances during the course of lung cancer and chemotherapy awaits elucidation. Peripheral blood mononuclear cells (PBMC) can be obtained with minimal invasive procedures; they represent peripheral tissues as a target for chemotherapy effects, have metabolic pathways present also in the nervous system (e.g., glutamate pathways), in pathological conditions penetrate central nervous system through blood-brain barrier, and cause local effects [15].

Neurotransmitters, including glutamate, mediate lymphocyte stimulation and production of cytokine [16] and in effect can orchestrate the remote effects of cancer on CNS. Thus, PBMC, which contain a fraction of immune cells, can be used as a model for the studies on the influence of cancer and/or chemotherapy on glutamate metabolism.

Glutamate-glutamine pathway, both in nervous system and in lymphocytes, delivers glutamate. Its production is controlled by glutaminase (GLS), while glutamate dehydrogenase (GDH) and glutamate decarboxylase catalyze its degradation [17, 18].

The aim of this prospective study was the analysis of glutamate content and activities of the enzymes, which control its metabolism in PBMC and serum in relation to neurological complications in lung cancer patients who are treated with chemotherapeutics.

## 2. Material and Methods

### 2.1. Patients

The prospective study included 221 lung cancer patients (152 males aged 62.1 ± 7.8 years and 69 females aged 62.6 ± 5.9 years, mean ± SD) hospitalized in the Sub-Department of Diurnal Chemotherapy Wielkopolska Center of Pulmonology and Thoracosurgery of Eugenia and Janusz Zeyland and in Department of Oncology, Poznan University of Medical Sciences. Among 221 patients 25 had small-cell lung cancer, 85 had adenocarcinoma, 62 had squamous cell carcinoma, 4 had large cell carcinoma, and 43 were NOS (not otherwise specified). Small-cell lung cancer patients underwent chemotherapy with carboplatin-etoposide or cisplatin-etoposide; adenocarcinoma patients underwent chemotherapy with pemetrexed-cisplatin or cisplatin-vinorelbine or cisplatin-gemcitabine or carboplatin-gemcitabine or erlotinib; squamous cell carcinoma patients underwent chemotherapy with cisplatin-vinorelbine or carboplatin-gemcitabine or carboplatin-vinorelbine or carboplatin-gemcitabine; large cell carcinoma patients underwent chemotherapy with cisplatin-etoposide or carboplatin-etoposide; NOS patients underwent chemotherapy with epidermal growth factor gene (EGFR) mutation-EGFR-tyrosine kinase inhibitors, and subjects without the mutation underwent chemotherapy with cisplatin-vinorelbine or carboplatin-vinorelbine or cisplatin-gemcitabine or carboplatin-gemcitabine. We enrolled in the study only patients who were not treated previously with chemotherapeutics.

All patients included in the study underwent neurological examination and clinimetric evaluation with the use of MiniMental State Examination (MMSE), Trail Making Test A and B (TMT A & B), and Hamilton scale at baseline (time of lung cancer diagnosis) and after 6-month follow-up. The intensity of symptoms in patients with neuropathy/polyneuropathy was evaluated with Katzenwadel scale [19]. Moreover, we used the Psychology Experiment Building Language (PEBL) software for objective evaluation of cognitive functions. It included Digit Span (DSpan) test and simple reaction time (SRT) and choice reaction time (CRT) tests. Working memory was evaluated by means of DSpan test. SRT evaluates the capacity of reactions to single stimulus. During this test, which is performed in 4 blocks of 50 trials with a break between blocks, a single stimulus appears at a specifiable delay (250 to 2500 ms) after the previous response. CRT is a reaction time to multiple stimuli. The PEBL tests were performed at baseline and after 6 months of chemotherapy. The results of PEBL tests performed in lung cancer patients were compared to reference values we have obtained from the control group of 19 age-matched (56 ± 11 years, *P* > 0.05) healthy subjects.

All patients underwent head computerized tomography with contrast to exclude brain metastases.

The study protocol was accepted by the Ethics Committee of Poznan University of Medical Sciences and each recruited participant gave written informed consent.

### 2.2. Onconeural Antibodies

We have analyzed patients' sera for the presence of onconeural antibodies and anti-neural antibodies by means of indirect immunofluorescence (EUROIMMUN, Germany) as a screening and line blot as confirmation test (EUROIMMUN, Germany). Indirect immunofluorescence used monkey cerebellum, monkey peripheral nerve, pancreas, and intestine as substrates and enabled the evaluation of the following:

1. Well-defined onconeural antibodies: anti-Hu, anti-Yo, anti-Ri, anti-CV2, anti-Ma/Ta, and anti-amphiphysin
2. Anti-neural antibodies: anti-MAG (myelin-associated glycoprotein), anti-myelin, anti-GFAP (glial fibrillary acidic protein), anti-GAD (glutamic acid decarboxylase), and anti-neuroendothelium
3. Anti-nucleosome antibodies: antibodies targeting nuclei in at least two tissues (e.g., the cerebellum and pancreas or cerebellum and intestine)

Well-defined onconeural antibodies were detected by means of a two-step method (indirect immunofluorescence followed by a line blot), while anti-neural antibodies and anti-nucleosome antibodies were evaluated by means of indirect immunofluorescence, because there are no recombinant antigen-based tests available, which can be used for confirmation.

### 2.3. Peripheral Blood Mononuclear Cells Isolation

Peripheral blood mononuclear cells (PBMC) were isolated from heparinized blood by density gradient centrifugation (Histopaque, Sigma-Aldrich). The isolated fractions were supplemented with protease inhibitors cocktail (Sigma-Aldrich). PBMC fractions were portioned and immediately stored at −80°C until analysis.

### 2.4. Biochemical Analyses

#### 2.4.1. Glutaminase Activity

Glutaminase activity (GLS) was measured basing on the evaluation of NH_3_ released from L-glutamine [20]. Incubation mixture contained 0.04 M L-glutamine (Sigma-Aldrich) in 0.1 M phosphate buffer pH = 7.0 and serum samples and was incubated in 37°C for 30 minutes. The reaction was stopped by adding 1.5 M trichloroacetic acid (TCA) (Sigma-Aldrich). After centrifugation 5000 ×g for 3 minutes of samples Nessler reagent (Sigma-Aldrich) was added to the supernatant, and the mixture was further incubated in room temperature for 10 minutes. The absorbance was measured at 450 nm in microplate reader ELx800 (Bio-Tek). Blank samples contained water, TCA, L-glutamine, and Nessler reagent. The standard curve was prepared with the use of (NH_4_)_2_SO_4_ as a standard. One unit of glutaminase was defined as an amount of the enzyme which released one micromol of ammonia. Specific activity in PBMC was expressed in milliunits per milligram of protein and its serum activity in milliunits per liter.

#### 2.4.2. Glutamate Dehydrogenase Activity

Glutamate dehydrogenase activity (GDH) was evaluated with the use of colorimetric kit (Abcam). The test is based on the consumption of glutamate by GDH in a sample and stoichiometrical generation of NADH, which causes a proportional color development. Specific GDH activity in PBMC was expressed in milliunits per milligram of protein and its activity in serum was expressed in milliunits per milliliter.

#### 2.4.3. Glutamate Decarboxylase Activity

Glutamate decarboxylase activity (GAD) was analyzed by means of colorimetric microplate method described by Yu et al. [21] in which bromocresol blue was used as pH indicator for GAD activity. The reaction mixture consisted of acetate buffer (20 mM, pH 4.8), 70 *μ*M bromocresol green, 10 mM pyridoxal 5′-phosphate, 2 *μ*L glutamate (from a 1 M stock in 20 mM acetate buffer, pH 4.8), and 5 *μ*L PBMC extract or 50 mL of serum. The change in absorbance at 620 nm was monitored at 40°C. The GAD activity was calculated as described previously [21] and expressed in units per milligram of protein in PBMC and in units per liter in serum.

#### 2.4.4. Glutamate

Free glutamate was evaluated with enzymatic colorimetric assay Kit (Abcam). Its content in PBMC was expressed in nanomole per milligram of protein and serum concentration in micromole per liter.

#### 2.4.5. Total Protein Analysis

Protein concentration in PBMC fraction was evaluated by means of Lowry method [22].

### 2.5. Statistics

Statistical analyses were performed using licensed MedCalc software.

## 3. Results

At baseline we have observed neurological deficit in 72% of lung cancer patients, of which 63% manifested symptoms from peripheral nervous system and 9% manifested CNS symptoms. Sensory neuropathy and sensorimotor neuropathy dominated among peripheral nervous system symptoms and cerebellar syndrome in the group of patients with CNS manifestations. After 6 months of chemotherapy 68% of lung cancer patients showed new symptoms. Sensorimotor neuropathy and cerebellar syndrome were the most frequent. We observed increase (*P* < 0.0001) in the intensity of neuropathy symptoms scored according to Katzenwadel scale after 6 months of chemotherapy (3.1 ± 1.5; mean ± SD) compared to baseline evaluation (1.8 ± 1.2).

The measures of executive functions after 6-month follow-up showed worsening (*P* < 0.05) in TMT A (8.0; 6.0–10.31; median; minimum–maximum) and TMT B (143.0; 118.75–172.5) compared to baseline times (TMT A: 6.83; 5.79–11.00 and TMT B: 123.38; 96.75–161.0, resp.). No changes in MMSE scores and Hamilton scale were found.

Cognitive function evaluation by means of The Psychology Experiment Building Language (PEBL) tests showed working memory impairment in Digit Span test in lung cancer patients at baseline (4.00; 3.64–4.44; median; minimum–maximum; all data below are presented in such a format; *P* < 0.0001) and after 6 months of chemotherapy (4.00; 3.64–4.36; *P* < 0.0001) compared to healthy controls (5.52; 4.56–6.22).

**Figure 1:**
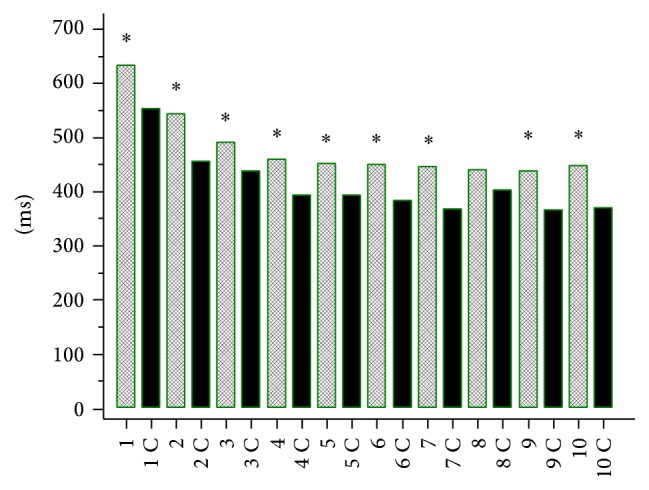
Simple reaction time (SRT) in lung cancer patients at baseline and in healthy controls (C). 1: 250 ms, 1 C: 250 ms control, 2: 500 ms, 2 C: 500 ms control, 3: 750 ms, 3 C: 750 ms control, 4: 1000 ms, 4 C: 1000 ms control, 5: 1250 ms, 5 C: 1250 ms control, 6: 1500 ms, 6 C: 1500 ms control, 7: 1750 ms, 7 C: 1750 ms control, 8: 2000 ms, 8 C: 2000 ms control, 9: 2250 ms, 9 C: 2250 ms control, 10: 2500 ms, and 10 C: 2500 ms control. The times [ms] indicate delay (250 to 2500 ms) after the previous response in SRT test. ^*∗*^ *P* < 0.05.

**Figure 2:**
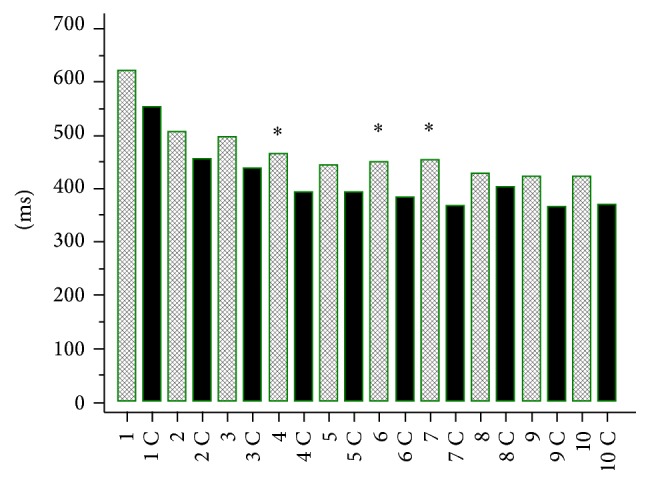
Simple reaction time (SRT) in lung cancer patients after 6 months of chemotherapy and in healthy controls (C). 1: 250 ms, 1 C: 250 ms control, 2: 500 ms, 2 C: 500 ms control, 3: 750 ms, 3 C: 750 ms control, 4: 1000 ms, 4 C: 1000 ms control, 5: 1250 ms, 5 C: 1250 ms control, 6: 1500 ms, 6 C: 1500 ms control, 7: 1750 ms, 7 C: 1750 ms control, 8: 2000 ms, 8 C: 2000 ms control, 9: 2250 ms, 9 C: 2250 ms control, 10: 2500 ms, and 10 C: 2500 ms control. The times [ms] indicate delay (250 to 2500 ms) after the previous response in SRT test. ^*∗*^ *P* < 0.05.

**Figure 3:**
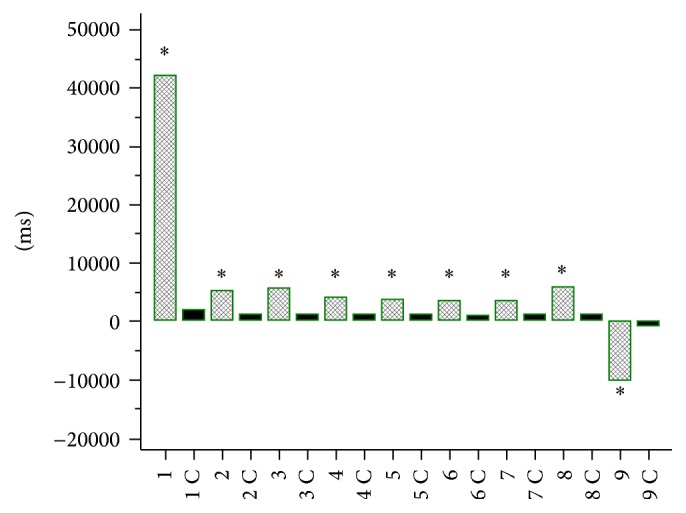
Choice reaction time (CRT) in lung cancer patients at baseline and in controls (C). 1, 2, 3, 4, 5, 6, 7 – CRT, 8 – mean CRT, 9 – the difference between CRT 7 and CRT 1 in lung cancer patients at baseline 1C, 2C, 3C, 4C, 5C, 6C, 7C – CRT, 8C – mean CRT, 9C – the difference between CRT 7C and CRT 1C in controls. ^*∗*^ *P* < 0.0001.

**Figure 4:**
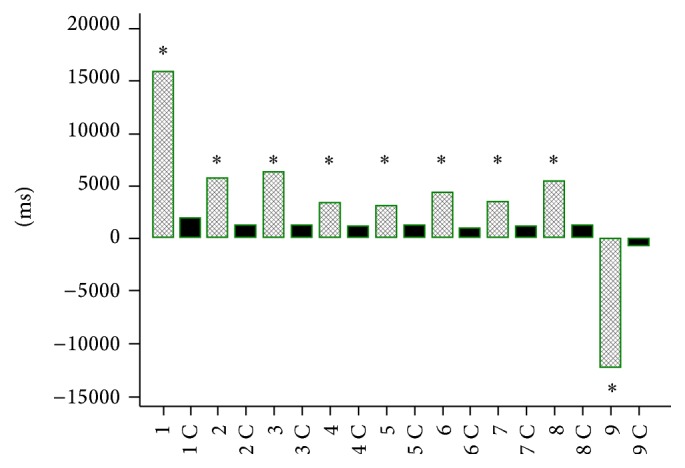
Choice reaction time (CRT) in lung cancer patients after 6 months of chemotherapy and in controls (C). 1, 2, 3, 4, 5, 6, and 7: CRT, 8: mean CRT, 9: the difference between CRT 7 and CRT 1 in lung cancer patients at baseline; 1C, 2C, 3C, 4C, 5C, 6C, and 7C: CRT, 8C: mean CRT, and 9C: the difference between CRT 7C and CRT 1C in controls. ^*∗*^ *P* < 0.0001.

Simple reaction time (SRT) (Figures 1 and 2) and choice reaction time (CRT) (Figures 3 and 4) were worse in lung cancer patients both at baseline and after 6 months of follow-up than in healthy controls.

We have found autoantibodies in 36% of lung cancer patients (seropositive patients) by means of indirect immunofluorescence. In the group of seropositive subjects 20% had anti-neural antibodies and 18% anti-nucleosome antibodies. By means of line blot we have confirmed the presence of onconeural antibodies (anti-Hu, anti-Ma/Ta) in 11% of lung cancer patients with anti-neural antibodies. In the spectrum of anti-nucleosome antibodies we have identified by means of line blot the following antibodies: RNP A, Sm, anti-centromere B, SS-A, Ro-52, anti-ds-DNA, and Scl-70. In the group of seropositive patients 14% had double antibodies (anti-nucleosome + anti-glial fibrillary acid protein; anti-neuroendothelium + anti-glial fibrillary acid protein; anti-nucleosome + anti-myelin).

Chemotherapy caused the increase in glutamate content in PMBC and elevation of its serum concentration, decrease in glutaminase activity in PBMC, and increase of glutamate dehydrogenase activity in PBMC (Tables 1 and 2). No effect on glutamate decarboxylase in PBMC and serum, as well on GDH and glutaminase in serum, was observed (Tables 1 and 2).

Glutamate concentrations in sera of lung cancer patients correlated negatively with working memory evaluated with DSpan test at baseline (*r*_S _ = −0.253; *P* = 0.05).

Glutaminase activity in PBMC negatively correlated with DSpan test score (*r*_S_ = −0.362; *P* < 0.001).

Baseline glutamate decarboxylase activity in PBMC negatively correlated with initial MMSE score (*r*_S_ = −0.360; *P* < 0.0001); similarly GAD activity in sera of lung cancer patients was related to impaired cognition evaluated by means of MMSE (*r*_S_ = −0.355; *P* = 0.0003). GAD activity in PBMC negatively correlated with DSpan (*r*_S_ = −0.362; *P* = 0.0004) at baseline.

Glutamate dehydrogenase activity in PBMC negatively correlated with MMSE score (*r*_S_ = −0.581; *P* = 0.0046), while serum GDH correlated positively with baseline MMSE (*r*_S_ = 0.778; *P* = 0.0392). Baseline GDH activity in PBMC correlated negatively with DSpan evaluated after 6 months of chemotherapy (*r*_S_ = −0.766; *P* < 0.001).

After 6 months of chemotherapy serum GDH activity correlated positively with TMT B (*r*_S_ = 0.919; *P* < 0.01).

Serum glutamate concentrations correlated positively with SRT test (correlation coefficients ranged from* r*_S_ = 0.288, *P* = 0.0311 for short delays, to* r*_S_ = 0.266; *P* = 0.0476 for longer delays in the test) at baseline and negatively after 6 months of chemotherapy (correlation coefficients ranged from* r*_S_ = −0.583, *P* = 0.0227 for short delays, to* r*_S_ = −0.583, *P* = 0.0227 for longer delays in the test). Serum glutaminase activity correlated negatively with SRT (correlation coefficients ranged from* r*_S_ = −0.350, *P* = 0.0082 for short delays, to* r*_S_ = −0.298, *P* = 0.0259 for longer delays in the test) at baseline and with CRT at baseline (*r*_S_ = −0.339, *P* = 0.0197). GAD activity in PBMC correlated positively with CRT (*r*_S_ = 0.209, *P* = 0.0351).

Serum glutamate concentrations were increased in lung cancer patients with neurological deficit present at baseline (401.03; 254.29–3484.48 *μ*mol/L; *P* < 0.05) compared to asymptomatic patients (457.88; 335.38–4664.32 *μ*mol/L). Baseline serum GDH activity was lower in patients manifesting neurological symptoms (84.06; 25.73–149.92 mU/mL, *P* < 0.05) than in patients without neurological deficit (107.67; 47.16–154.88 mU/mL). Patients with CNS symptoms had higher serum GAD activity (444.01; 218.11–926.97 U/L; *P* < 0.05) than subjects with peripheral nervous system symptoms at baseline (366.11; 46.73–817.92 U/L).

New neurological symptoms observed after 6 months of chemotherapy were associated with higher GAD activity in PMBC (213.0; 125.0–308.0 U/mg protein; *P* < 0.01) than in patients without neurological complications of the treatment (156; 76–176 U/mg protein).

In seropositive lung cancer patients serum GDH activity was lower (70.96; 41.8–109.65 mU/mL; *P* < 0.05) than in seronegative subjects (99.93; 74.34–130.68 mU/mL).

Serum GDH activity was lower in squamous cell lung cancer (84.06; 25.73−126.91 mU/mL) than in small-cell lung cancer (96.36; 40.41–149.92 mU/mL; *P* = 0.027) and in large cell lung cancer (99.93; 73.54−134.05 mU/mL; *P* = 0.007).

## 4. Discussion

In our study we have found that lung cancer as well as chemotherapy affects glutamate metabolism. The changes of glutamate levels and activities of enzymes that control glutamate metabolism are more pronounced in PBMC than in serum. The clinimetric measures of neurological deficit and cognitive functions in lung cancer patients correlated mainly with changes in glutamate levels and activities of glutamate degrading enzymes, that is, GDH and GAD. The modifications of activity of glutamate synthetizing enzyme, glutaminase, were less important, but noticeable.

Glutamine-glutamate metabolism dysregulation was already observed in cancer. It develops as a consequence of glycolysis upregulation known as “Warburg effect” [23]. Glutamine serves in this process as a source of carbon for further anabolic pathways. Transcriptional and posttranslational modifications of glutamine-glutamate metabolism controlling enzymes can lead in some types of cancers to “glutamine addiction,” which is considered as a new therapeutic target [24]. Interestingly, aberrant expression of methylated glutamate type 2B NMDA receptor (N-methyl-D-aspartate receptor) was identified in non-small-cell lung cancer, especially in squamous cell lung cancer, as a molecular marker [25].

In our study we observed decreased serum GDH activity in squamous cell lung cancer patients comparing to small-cell lung cancer and large cell cancer. In small-cell lung cancer patients xc-cystine/glutamate antiporter was reported as a potential therapeutic target for sulfasalazine [26]. Thus, the studies on glutamate metabolism in lung neoplasms seem to be promising in terms of future therapies.

However, less is known about glutamate metabolism in peripheral tissues of lung cancer patients. Glutamate receptors and transporters were found in T and B lymphocytes, dendritic cells [27], and macrophages [28], all of which are present in the fraction of peripheral blood mononuclear cells. The activation of dendritic cells is associated with glutamate production [29]. The stimulation of *α*-amino-3-hydroxy-5-methyl-4-isoxazolepropionic acid receptor (AMPA receptor) by glutamate leads to activation and migration of T lymphocytes [30], which can penetrate central nervous system.

In our study we have observed that humoral immune response during the course of lung cancer was associated with lowered serum GDH activity. The presence of anti-neural or onconeural antibodies was not related to the changes of glutamate metabolism markers we have analyzed. There are only limited data on GDH activity relations to autoimmunity or lymphocytes. In animal model of experimental allergic encephalomyelitis GDH is downregulated in astrocytes [31]. On the other hand it was shown that the proliferation of B cells leads to intracellular upregulation of GDH activity [18]. Thus, glutamate metabolism requires further studies in patients with neurological paraneoplastic syndromes (PNS), which are considered as immune-mediated remote effects of cancer. However, some PNS patients are seronegative and non-immune-mediated pathomechanisms in cases like cerebellar degeneration can be taken into consideration.

We have found increase of serum glutamate concentrations in lung cancer patients with neurological deficit and upregulation of serum GAD activity in subjects with CNS symptoms, particularly with cerebellar syndrome, while serum GDH activity was downregulated in patients manifesting neurological symptoms.

Increased plasma glutamate concentrations were found in olivopontocerebellar atrophy associated with GDH deficiency [32]. In the present study we can also explain serum glutamate level elevation by downregulation of GDH activity in lung cancer patients, who manifest neurological deficit.

Experimental studies provided different data on CNS lesions and GAD activity. The injury of climbing fibers in cerebellum caused upregulation of GAD activity in the vermis [33]. Neurotoxic damage of inferior olive-climbing fiber projection to Purkinje cells in the cerebellum stimulated GAD activity in Purkinje cell axons endings in the deep cerebellar nuclei [34]. On the other hand, impairment of GABA-ergic transmission in cerebellum was found after GAD inhibition, which also was associated with increase in the number of natural killer cells and their cytotoxicity [35]. Immunological inhibition of GAD activity decreases glutamate degradation leading to hyperexcitability and opsoclonus as its clinical manifestation [36]. Thus, increased serum GAD activity in our study can result from CNS lesions during the course of lung cancer.

Decreased GDH activity in leukocytes was reported in patients with spinocerebellar ataxia and extrapyramidal syndromes [37, 38]. In the light of such data one may propose the hypothesis on the role of GDH inhibition in cerebellar degeneration during the course of lung cancer. Moreover, we noticed that downregulation of GDH activity in PBMC was related to impaired cognition in lung cancer patients.

Glutamate metabolism dysregulation we have observed in lung cancer patients can be linked to cognitive impairment, because it causes imbalance between excitatory neurotransmitter and *γ*-aminobutyric acid (GABA), which is an inhibitory agent. Such a hypothesis is supported by studies performed in experimental animals [39] and in human brains [40]. Moreover, immunological inhibition of GAD activity in diabetes patients was associated with cognitive impairment [41].

Six-month chemotherapy of lung cancer induced in our study the changes in glutamate expression and glutamate synthetizing enzyme as well as degrading enzyme in PBMC. Key regulatory enzymes of glutamine-glutamate pathways and their metabolites participate in chemotherapy-induced neurological complications. Clinical observation suggests the protective role of glutamine against neurotoxicity induced by chemotherapeutics, for example, paclitaxel [42] or oxaliplatin [43]. However, conflicting data are provided by experimental and clinical studies on safety and effectiveness of glutamine supplementation in oncological patients treated with chemotherapeutics [44, 45]. Inhibition of glutamate carboxypeptidase, which catalyzes hydrolysis of N-acetyl-aspartyl-glutamate (NAAG) into N-acetyl-aspartyl (NAA) and glutamate, protects against neuropathy induced by cisplatin, paclitaxel, and bortezomib [46]. Moreover, downregulation of glutamate carboxypeptidase decreases the risk of chemotherapy-induced or diabetic neuropathy [47]. Recently, glutamate is considered as possible agent involved in pathomechanisms leading to neuropathy [48].

To conclude, dysregulation of glutamate metabolism in PBMC develops in lung cancer patients after chemotherapy. Changes in glutamate level and in activities of glutamate synthetizing and degrading enzymes are related to neurological deficits, including cognitive impairment observed as remote effects of lung cancer and its chemotherapy. Markers of glutamate pathways can be a starting point for the support of diagnostics, monitoring, prediction, and the development of therapeutic strategies in lung cancer patients with neurological complications.

## Figures and Tables

**Table 1:** The content of glutamate and activities of GLS, GDH, and GAD in PBMC of lung cancer patients during chemotherapy. ^*∗*^ *P* < 0.01; ^+^ *P* < 0.05, and ^#^ *P* < 0.001.

	Baseline	After 6-month chemotherapy
Glutamate (nmol/mg protein) median; minimum–maximum	1.03 0–5543.42	253.5^*∗*^ 0–41615.30

Glutaminase (mU/mg protein) median; minimum–maximum	0.42 0–91.33	0.089^+^ 0–31.97

Glutamate dehydrogenase (mU/mg protein) median; minimum–maximum	3.67 1.57–1594.61	15.99^#^ 4.08–3185.02

Glutamate decarboxylase (U/mg protein) median; minimum–maximum	0.85 0–33.25	0.93 0–79.98

**Table 2:** The concentration of glutamate and activities of GLS, GDH, and GAD in sera of lung cancer patients during chemotherapy. ^*∗*^ *P* < 0.01.

	Baseline	After 6-month chemotherapy
Glutamate (*μ*mol/L) median; minimum–maximum	412.83 85.41–5456.27	3971.83^*∗*^ 399.74–4937.04

Glutaminase (mU/L) median; minimum–maximum	1.66 0–3.02	0.63 0.33–2.61

Glutamate dehydrogenase (mU/mL) median; minimum–maximum	89.81 25.73–154.88	105.78 66.60–134.65

Glutamate decarboxylase (U/L) median; minimum–maximum	405.06 0–1589.09	611.49 210.32–903.60
